# Draft Genome Sequence of *Kluyveromyces marxianus* Strain DMB1, Isolated from Sugarcane Bagasse Hydrolysate

**DOI:** 10.1128/genomeA.00733-14

**Published:** 2014-07-24

**Authors:** Toshihiro Suzuki, Tamotsu Hoshino, Akinori Matsushika

**Affiliations:** a Biomass Refinery Research Center, National Institute of Advanced Industrial Science and Technology, Higashihiroshima, Hiroshima, Japan; b Graduate School of Advanced Sciences of Matter, Hiroshima University, Higashihiroshima, Hiroshima, Japan

## Abstract

We determined the genome sequence of a thermotolerant yeast, *Kluyveromyces marxianus* strain DMB1, isolated from sugarcane bagasse hydrolysate, and the sequence provides further insights into the genomic differences between this strain and other reported *K. marxianus* strains. The genome described here is composed of 11,165,408 bases and has 4,943 protein-coding genes.

## GENOME ANNOUNCEMENT

*Kluyveromyces marxianus* is a thermotolerant yeast that shows ethanol productivity from glucose similar to that of *Saccharomyces cerevisiae* at 30°C (1) and can survive at 52°C (2). *K. marxianus* can utilize various hexose and pentose sugars, including xylose, xylitol, and xylulose, and can ferment xylose at a high temperature (1, 3). Therefore, an attempt has been made to use *K. marxianus* for simultaneous saccharification and fermentation for direct conversion of lignocellulosic biomass to ethanol (4). *K. marxianus* DMB1 was isolated from a hydrolysate derived from sugarcane bagasse as a novel thermotolerant yeast (5). *K. marxianus* DMB1was classified into the *K. marxianus* NBRC1777 clade by D1/D2 and internal transcribed spacer sequence analysis (5). However, *K. marxianus* DMB1 was more thermotolerant than NBRC1777 and produced ethanol at 48°C (5). Furthermore, *K. marxianus* DMB1 can utilize sorbitol, which is not assimilated by NBRC1777 (5). To better understand the differences in gene sequences and phenotypes among *K. marxianus* strains and to use basic genetic engineering techniques, we determined the draft genome sequence of *K. marxianus* DMB1.

Genomic DNA was extracted using the MasterPure Yeast DNA purification kit (Epicenter) and purified using NucleoBond AXG 100 and NucleoBond buffer set III (Macherey-Nagel) according to the manufacturers’ instructions. The purity and concentration of DNA were measured by NanoDrop (Thermo Scientific) and the Quant-iT dsDNA BR assay kit (Invitrogen). The genomic DNA (5 µg) of DMB1 was fragmented to approximately 10-kb pieces using g-TUBE (Covaris, Inc.), and SMRTbell libraries were prepared by using DNA template prep kit version 2.0 (3 kb to 10 kb) (Pacific Biosciences). The concentration was measured by using the Quant-iT dsDNA BR assay kit, and library size analysis was performed using Agilent 2200 TapeStation (Agilent Technologies, Inc.). SMRTbell libraries were bound to polymerases using the DNA/Polymerase binding kit P40 (Pacific Biosciences). Calculation of the concentration of polymerase-template complex for binding and annealing reaction was performed by using Binding Calculator version 2.1.0.1 (Pacific Biosciences). These complexes were bound to magbeads using the MagBead kit (Pacific Biosciences) and loaded on a total of 16 SMRT cells (SMRT Cells 8Pac version 3; Pacific Biosciences). The sequence reaction was performed on a PacBio RS II (Pacific Biosciences). Raw data from the 16 SMRT cells were 35 million reads at 312-fold coverage and were assembled *de novo* using SMRT Analysis version 2.1.1 (Pacific Biosciences) (6) to filter subreads and circular consensus sequence reads. The genome sequence of DMB1 was 11,165,408 bases, and the GC content was 40.1%. The assembly generated 36 contigs with a maximum length of 1,733,117 bases and an *N*_50_ length of 1,416,405 bases. We performed prediction of the open reading frames and proteins with MAKER2 version 2.10 (7), AUGUSTUS version 3.0 (8), and NCBI BLAST version 2.2.20 (9) and found 4,943 putative open reading frames similar to sequences of strains of *Kluyveromyces lactis*. We also identified 212 putative tRNA genes using tRNAscan version 1.23 (10).

### Nucleotide sequence accession numbers.

The nucleotide sequence of the *Kluyveromyces marxianus* DMB1 draft genome has been deposited in DDBJ/EMBL/GeneBank under the accession numbers BBIL01000001 to BBIL01000036. The version described in this paper is the first version.
